# Cross-Cultural Adaptation and Validation of the Romanian Marx Activity Rating Scale for Anterior Cruciate Ligament Reconstruction

**DOI:** 10.3390/healthcare8030318

**Published:** 2020-09-04

**Authors:** Oana Suciu, Radu Prejbeanu, Horia Haragus, Cosmin Faur, Roxana Ramona Onofrei, Adrian Todor

**Affiliations:** 1 Department of Rehabilitation, Physical Medicine and Rheumatology, ‘Victor Babes’ University of Medicine and Pharmacy Timisoara, No 2 Eftimie Murgu Square, 300041 Timisoara, Romania; oanasuciu78@umft.ro (O.S.); onofrei.roxana@umft.ro (R.R.O.); 2 Department of Orthopedics and Trauma, ‘Victor Babes’ University of Medicine and Pharmacy Timisoara, No 2 Eftimie Murgu Square, 300041 Timisoara, Romania; raduprejbeanu@gmail.com (R.P.); faur17@gmail.com (C.F.); 3 Department of Orthopedics, Traumatology and Pediatric Orthopedics, “Iuliu Hatieganu” University of Medicine and Pharmacy, No 8 Victor Babes Str, 400000 Cluj-Napoca, Romania; adi.todor@yahoo.com

**Keywords:** Marx activity rating scale, anterior cruciate ligament, anterior cruciate ligament reconstruction/rehabilitation, athletic injuries, outcome assessment, health care

## Abstract

Aim: We aimed to translate, cross-cultural adapt and validate the Marx activity rating scale (MARS) of the knee for Romanian patients with anterior cruciate ligament (ACL) injury. Method: The original English form was translated according to guidelines. We included patients with ACL injury undergoing reconstruction in two centers over 3 years. Subjects completed the translated MARS, International Knee Documentation Committee (IKDC) subjective knee form and EuroQol EQ5D. The examining physician completed the Tegner Lysholm scale as an objective evaluation. Re-testing was obtained after one month. We used Spearman`s correlation to evaluate construct validity and reproducibility, Cronbach’s alpha for internal consistency and intraclass correlation for test-retest reliability. Results: We collected valid forms from 99 patients (32.1 ± 8.8 years, 64.6% males) during the preoperative evaluation and 45 were re-tested. Significant, very good correlations were found between the MARS and Tegner Lysholm (Spearman’s r = 0.712, *p* < 0.0001) and IKDC (Spearman’s r = 0.801, *p* < 0.0001). Cronbach’s alpha was 0.893 at the initial completion and 0.799 at re-test. The intraclass correlation coefficient was 0.895. Conclusions: The Romanian-translated MARS is a valid, consistent and reliable physical activity outcome measure in patients with anterior cruciate ligament reconstruction.

## 1. Introduction

Anterior cruciate ligament (ACL) injury is one of the most frequent pathologies of the knee, especially in the young athletic population. It is the main stabilizer against anterior tibial translation, with functional importance in sports [1,2]. ACL reconstructions are increasing, with predictions for our country to exceed 3000/year for state insurance reimbursements alone [1,3].

In the last few decades, patient reported outcome (PROs) measures have become more and more important in order to complete the physician’s examination and to better measure issues important to patients. Among the most commonly used evaluations for ACL reconstruction are the subjective International Knee Documentation Committee (IKDC), Tegner Lysholm, Knee Disability and Osteoarthritis Outcome Score (KOOS), Marx activity rating scale (MARS) and EuroQol EQ5D scores together with KT1000™ (MEDmetric^®^ Corporation, San Diego, CA, USA) for anterior translation and clinical pivot-shift test [4,5,6].

The MARS is a validated knee-specific patient-reported outcome measure, which reliably assesses the frequency of participation in sports-related activities for an adult population. The MARS was developed and published in 2001 as an alternative to the Tegner activity score. Unlike the Tegner scale, the MARS is not based on participation in specific sporting activities as it evaluates different components of physical function that are common in various sports [7,8,9].

Activity level is an important aspect that should be evaluated separately, thus the need for scales such as MARS [7,8,9]. Frequency and intensity of sports participation varies widely among patients. As a consequence, a way of quantifying this activity is critical for studies comparing two treatment outcomes to ensure that the patient groups are equivalent with respect to this characteristic [9]. Patients with a knee ligament injury may report higher MARS scores but lower KOOS and Western Ontario and McMaster Universities Osteoarthritis Index (WOMAC) scores after injury. There are also some cases in which patients may have good pain and function outcomes but low activity scores, which indicates the importance of including an activity rating instrument in the postinjury assessment of patients. [10]. Current medical literature suggests that the MARS is a reliable and valid patient reported outcome measure for assessing sports-related activities. It has been translated and culturally adapted in several languages but not in Romanian [7,11,12].

We therefore aimed to perform the translation, cross-cultural adaptation and validation of the MARS in patients with anterior cruciate ligament reconstruction.

## 2. Materials and Methods

### 2.1. Translation

The MARS is composed of 4 questions asking about running, changing directions while running, decelerating and pivoting during sports in the healthiest, most active state in the past year. The answers are scored on a 5-point scale from 0: less than once a month to 4: more than 4 times a week, with a total score ranging from 0 to 16 [9]. The English (US) version was translated and culturally adapted following the principles of good practice for the translation and cultural adaptation of the International Society for Pharmacoeconomics and Outcomes Research (ISPOR) [13]. Two independent native Romanian speaker translators translated the English version into Romanian. Afterwards the two forms were reviewed by the authors together with the translators and a common form was decided with more conceptual equivalence. A backward translation from Romanian to English of the last form was made by one native English speaker. A committee formed by the authors and translators compared the backward translation with the original English (US) MARS form. For pre-testing two authors interviewed 5 subjects in order to correct any confusing items or other semantic problems.

### 2.2. Enrollment

Subjects were screened from the cases that were examined during clinics and had an ACL injury as the main complaint. Diagnosis of ACL injury was made by the treating physician (orthopedic surgeon) using patient history, clinical examination, magnetic resonance imaging (MRI) and arthroscopic exploration (where available). Indication for surgical treatment and reconstruction were based on current guidelines using a standard anatomic single bundle technique [1,14]. Inclusion criteria were: acute or chronic primary ACL tear with or without medical collateral ligament (MCL) meniscus and cartilage injury as chief complaint. Exclusion criteria were: noncompliance, revisions, multiple ligament injuries, complex cases (ACL tear with tibial plateau fracture), large osteochondral fractures that require fixation, large focal cartilage defects requiring surgery, posterolateral corner, significant osteoarthritis, patients deemed to be without surgical indication/who would not benefit from reconstruction based on current guidelines, irrespective of their final decision. Partial ACL tears and cases with possible indication of repair were also excluded. For associated lesions, meniscal tears were determined during arthroscopic evaluation; no grading system was used for size, type and treatment (meniscectomy, suture or left as is: option for the lateral only) [15]. Cartilage damage was evaluated arthroscopically using the modified Outerbridge system: 0—normal, 1—softening, 2—superficial fissures under 1.5 square cm, 3—fissures deep to the subchondral bone, over 1.5 square cm, 4—exposed bone [16].

Patients completed the Romanian MARS, the Romanian IKDC subjective knee form and the Romanian EuroQol EQ5D5L [17,18]. The examining physician completed the Tegner Lysholm scale as an objective clinician evaluation [19]. Re-testing data were requested from the first patients who were available and agreed until we obtained over 40 valid and complete MARS forms after a target of one month. Raw data are available upon reasonable request from the corresponding author.

### 2.3. Ethics

The study was conducted in accordance with the Declaration of Helsinki and the protocol was approved by our local emergency clinical county hospital ‘Pius Brinzeu’ Timisoara Ethics committee for scientific research (no 180/2020). Patients gave their informed consent for inclusion before they completed the scores.

### 2.4. Questionairre Characteristics

The MARS evaluates four activity items: running, deceleration, cutting and pivoting. It has a 5-point scale referring to the frequency of performing these activities in the past 12 months, with a higher score meaning more frequent participation. The original MARS was created by consulting with orthopedic surgeons who specialized in sports medicine. It was designed to be completed in a 1 min period with four items identifying the level of activity and the frequency with which these activities are performed. Relevant items were established by interviewing a group of 20 patients with knee disorders and consulting with orthopedic surgeons, physical therapists and athletic trainers who specialized in sports medicine. The final form of four items was generated after interviewing a group of 50 patients with knee pathology, for its validity. The most important items and physically challenging to perform were chosen. The final form was determined based on both patient data and clinician opinion [7,8,9].

In the original study where the Marx activity rating scale was developed, convergent validity was evaluated by co-administering it with three other activity scales: Tegner, Cincinnati and Daniel [9]. A higher score indicates more functional demand on the knee joint and potentially a higher risk of injury. Preliminary data for the MARS suggest that it has good test-retest reliability (intraclass correlation coefficient, 0.97) and adequate concurrent and divergent validity [12]. The IKDC is one of the most commonly used and validated knee-specific outcome measures. The subjective knee form examines three dimensions: symptoms, sports activity, and knee function. EuroQol EQ5D5L is a simple 5 item scale and a visual analogue scale that examines overall health status. From these items an index can be computed using values predetermined for the target population (country), as well as quality-adjusted life years and disability-adjusted life years. The Tegner Lysholm scale is a measure of knee activity limitations. It consists of eight items that measure limp, need for support, pain, instability, locking, swelling, stair climbing and squatting.

### 2.5. Statistical Analysis

The validation process followed the COnsensus-based Standards for the selection of health Measurement INstruments (COSMIN) checklist [20]. To evaluate the construct validity, the Spearman’s correlation coefficient was used. A correlation between 0.41 and 0.6 was considered good, between 0.61 and 0.8 very good and a correlation coefficient greater than 0.81 was considered excellent. Content validity was determined by the presence of floor and ceiling effects: if more than 15% of patients had the lowest or the highest score. Internal consistency was tested by the Cronbach’s alpha coefficient at the first and second administration of the MARS. A Cronbach alpha value > 0.7 indicates a high level of internal consistency. The test-retest reliability was assessed by the intraclass correlation coefficient (ICC, two-way mixed effects model). An ICC greater than 0.75 was considered moderate, between 0.75 and 0.90 good, and values greater than 0.90 excellent. The reproducibility of the questionnaire was assessed using the Spearman’s correlation coefficient [17,20,21]. All data were tested for normality with the Shapiro–Wilk test. The data are presented as mean and standard deviation for normal distributed data, and as median and interquartile range for non-normally distributed data. Statistical significance was considered if *p* < 0.05. Analysis was done with the MedCalc software version 19.1.3 (MedCalc Software Ltd., Ostend, Belgium).

## 3. Results

### 3.1. Translation

In our project, most questions were considered simple and straightforward, with no content or form difficulties for translation. In accordance with previous authors, for item two ‘cutting’ was not translated to an equivalent word but instead adapted to a short explanatory construction (see Supplementary) [10,11]. Time required for answering was not recorded as we did not consider this aspect to be of any relevance for a four-item questionnaire developed to be completed in less than 1 min. 

### 3.2. Subjects

We screened 125 eligible patients from those planning to undergo ACL reconstruction in two centers, from two cities between 2017 and 2019. We collected valid and complete data sets from 99 patients (76/23 Cluj-Napoca/Timisoara, Romania) at the first administration of the questionnaire, usually during the preoperative evaluation. During our study period, 77 underwent surgery (all 45 from the re-test group). Re-testing data were collected from 45 patients at a mean of 4.8 weeks (range 2–8 weeks). Seven completed the form in the immediate postoperative period (at the first follow-up visit at two weeks). Patient demographics and associated lesions are presented in Table 1. Mean questionnaire results are presented in Table 2.

### 3.3. Validity

Significant, very good correlations were found between the MARS score and the Tegner Lysholm scale (Spearman’s r = 0.712, *p* < 0.001) and IKDC (Spearman’s r = 0.801, *p* < 0.001), respectively (Table 3). A tariff to compute the index is not yet available for Romania and therefore this was not included in the numerical comparison. For the overall MARS score there were no floor or ceiling effects, with 6.1% of patients reporting the lowest score (0) and 3% the highest score, respectively (16).

### 3.4. Reliability

The MARS questionnaire had a high degree of reliability. Cronbach’s alpha was 0.893 at the initial completion of the questionnaire and 0.799 for the re-test, respectively. The ICC was 0.895 (95% CI 0.804–0.943). The inter-item correlation matrix for the two assessments is presented in Table 4.

### 3.5. Reproducibility

The median MARS score reported at the first administration of the questionnaire was seven [3–12] and at the second administration it was five [3–9.25]. The two scores were strongly, positively and significantly correlated (Spearman’s r = 0.931, *p* < 0.0001).

## 4. Discussion

The Marx activity rating scale was successfully translated and cross-culturally adapted into Romanian. The adapted version has good reliability and validity to evaluate knee activity in patients who will undergo anterior cruciate ligament reconstruction.

The main clinical benefit is to be able to assesses knee-specific activity level, to determine the patient’s condition, lifestyle and engagement in sports. This can be instrumental when determining treatment indications such as ACL reconstruction as well as the outcome of the return to an active lifestyle and sport. As it is not disease-specific, the clinical usefulness can be extrapolated to determine the level of physical activity engagement resulted from any knee disorder. On the other hand, the scale only examines the physical activity status and does not include a psychological dimension that would consider fear of reinjury or lifestyle changes. As it evaluates strenuous and high-demanding movement, it is only appropriate for subjects that are fit and physically active. For sedentary subjects, those confined solely to activities of daily living or subjects who did not sustain their injury during a sporting activity, MARS will not be suitable to capture their knee-related physical impairment or gain [12].

In our project, all four questions were considered simple and straightforward, with no content or form difficulties for translation. This comes in contrast to Flosadottir et al. who changed the sports listed in item four, with the intent of covering Swedish sports with high rates of knee injuries: “Kicking, throwing, hitting a ball” to “ball sports/games” with the addition of the sample sports “soccer, team handball, floorball, basketball, and racquet sports”. Tennis and squash were condensed to racquet sports, and golf was excluded as it was considered to fall under the term of “ball sports/games” [12]. Flosadottir et al. also suggested an adjustable recall period, that is, one month or one week, as an option to one year in the English version to facilitate treatment follow-up [12]. Even though we admit ‘golf’ is seldom played in Romania, we left it as is in item four for two reasons: it was considered by the team to be easily understood by the general Romanian subjects even though it is not played, and its impact and influence on item 4 should be limited since there are sufficient other sporting activities listed, which would be better suited for Romanian population.

The test-retest reliability was acceptable with an ICC of 0.895 and a Cronbach’s alpha of 0.893 and 0.799 for the re-test, respectively. These values are in line with previous reports: ICC of 0.97 in the original study to develop the MARS, ICC of 0.78 for the Iranian and ICC > 0.9 and a Cronbach’s alpha >0.95 for the Swedish translation, respectively [9,10,12].

In our study, the test for floor and ceiling effects found fewer minimal and maximal responses (6.1% and 3%, respectively) compared to Flosadottir et al. (22% and 20%) and Negahban et al. (35%). In addition, Flosadottir et al. found the floor effect to differ considerably between participants who got injured during sporting activities (14.5%) and those during any other activity (59%) [11,12]. We therefore presume that this discrepancy may be related to participant lifestyles or demographics: mean age 32 versus 25 and 27% of males 64.6 versus 92 and 45, time from injury five months versus two and three [11,12].

The re-test patients were chosen arbitrarily. The lower value of the re-test MARS (median 5 [3–9.25] versus 7 [3–12] initially) could have been determined by having administered the forms after surgery for some patients, which is a limitation of our study. We did not record/account for the chronicity of the ACL tear, associated lesions (such as chondral damage), procedures performed concomitantly (such as meniscectomy/meniscal suture), body mass index or professional versus amateur athletes. Our main objective was to validate the translated MARS for future use in Romanian patients who will undergo ACL reconstruction.

In the original validation study, convergent validity was tested in co-administration with the Tegner, Cincinnati and Daniel Scales, demonstrating moderate or excellent correlation with all three of them (0.66, 0.67 and 0.52) [9]. In our study, we chose Tegner Lysholm, IKDC subjective form, and EQ5D because we expect these scales to be more relevant and used in current and future academic and research projects, and are also pertinent choices according to the reviewed literature [4,5,6,7,8]. MARS correlated very well with Tegner Lysholm (Spearman’s r = 0.712, *p* < 0.0001) and IKDC (Spearman’s r = 0.801, *p* < 0.0001). Negahban et al. found lower correlation coefficients but tested convergent validity against the individual subscales of KOOS and ShortForm-12 (SF-12). Flosadottir et al. reported correlations > 0.310 with KOOS subscales, Tegner and modernized Saltin–Grimby physical activity level scale [11,12].

Preinjury, current, and desired physical activity levels are assessed for both clinical and research purposes. Activity level is also used as a baseline characteristic when comparing athletic patients. We focused on the MARS because of its versatility for use in the physically active population, such as ACL reconstruction patients. Together with the IKDC subjective knee form they show good properties for orthopedic sports medicine knee conditions follow-up and return to play [7,8,12].

Return to sports and physical activity levels are major concerns, often used to evaluate surgical interventions and rehabilitation. Cox et al. showed that advanced cartilage lesions in the medial compartment and meniscal tears at the time of ACL reconstruction are predictive of poorer IKDC, KOOS and MARS scores after six years [22].

However, these do not exclude a physical evaluation including quadriceps strength and tests such as the single leg hop. It is also not specific to ACL and does not take in consideration the anxiety of future trauma, such as the anterior cruciate ligament-return to sport after injury questionnaire (ACL-RSI). This is one of the most used instruments in evaluating the psychological readiness of athletes for returning to sport after ACL reconstruction [23]. This 12-item questionnaire is significantly longer and emphasizes a single dimension, although a shorter version has also been proposed [24]. The most important aspect of this outcome measure is its ability to predict psychological withdrawal from intense physical activity. Compared to previously reported kinesiophobia scales with general addressability, the ACL-RSI was specifically developed to address ACL reconstruction patients and had proved a reliable predictive measure for resuming sports. It is focused on fear of reinjury, fear of repeating surgery, negative emotions, shifts in priority and individual personalities. While we acknowledge its usefulness, in our study we aimed to focus on an activity tool with wider applicability [23,24].

Survey length and time to collect data are perceived as very important factors of the burden associated with collecting patient reported outcomes [25]. A recent analysis demonstrated high correlation of the Patient-Reported Outcomes Information System Physical Function Computer Adaptive Test (PROMIS PF CAT) with commonly used scores such as Short Form-36 Physical Function (SF-36 PF), Knee Injury and Osteoarthritis Outcome Score (KOOS), Marx activity rating scale (MARS) and EuroQol EQ-5D. This system is also versatile and easy to administer, with a mean of 4.2 items (range 4–11) [8,26]. The presumed effect of computer-adaptive tests such as this is that they will be able to provide wide addressability. The possibility of using a single instrument to evaluate many dimensions of different pathologies from different anatomical areas with comparable efficacy to specific validated instruments and less burden for completion could be a game changing experience, which we look forward to exploring in the future.

When using PROs for scientific purpose in order to assess knee function and symptoms it is important to evaluate the patient’s level of sports activity along with the frequency with which they perform high demanding activities such as running or pivoting. The current literature suggests that the MARS is a reliable and valid PROs measures to assess the frequency of participation in sports-related activities for an adult population.

## 5. Conclusions

The Romanian translation of the activity rating scale is a valid, consistent and reliable outcome measure in patients with anterior cruciate ligament reconstruction.

## Figures and Tables

**Table 1:** Patient demographics and associated lesions.

	Test (*n* = 99)	Re-Test (*n* = 45)
Age (years, mean ± SD)	32.11 ± 8.87	29.71 ± 8.34
Gender (% males)	64.6%	68.9%
Time from injury (Months) Mean, SD, Range	5.1 ± 4.26; 2–24 (*n* = 63)	4.2 ± 3.57; 2–24 (*n* = 39)
Medial meniscus tear Lateral meniscus tear Both	45.5% (*n* = 77) 32.5% (*n* = 77) 16.9% (*n* = 77)	46.7% 33.3% 15.5%
Cartilage damage (0:1:2)	5.1:1:1.6 (*n* = 77)	4.8:1:1.6

**Table 2:** Questionnaire results.

	Test (*n* = 99)	Re-Test (*n* = 45)
MARS total score	7 [3–12]	5 [3–9.25]
IKDC	49.40 [40.20–62.92]	48.30 [35.60–57.77]
Tegner-Lysholm	66 [51–79]	-
EQ5D VAS	80 [65–90]	-

Table 2. Questionnaire results presented as median and interquartile range; Marx activity rating scale (MARS), International Knee Documentation Committee (IKDC) and EuroQol visual analogue scale (EQ5D VAS).

**Table 3:** Correlation between the tested scores.

	EQ5D VAS	Tegner-Lysholm	IKDC	MARS
EQ5D VAS	1.000	0.295 *	0.438 **	0.434 **
Tegner-Lysholm		1.000	0.848 **	0.712 **
IKDC			1.000	0.801 **
MARS				1.000

Table 3. Spearman’s correlation coefficient between the tested scores (*n* = 99) * *p* = 0.003; ** *p* < 0.0001; EuroQol visual analogue scale (EQ5D VAS), International Knee Documentation Committee (IKDC) and Marx activity rating scale (MARS).

**Table 4:** Inter-item correlation for the two MARS forms.

	**M1**	**M2**	**M3**	**M4**
M1	1.000	0.745	0.704	0.489
M2		1.000	0.840	0.670
M3			1.000	0.592
M4				1.000
	**M1 (re-test)**	**M2 (re-test)**	**M3 (re-test)**	**M4 (re-test)**
M1(re-test)	1.000	0.515	0.594	0.437
M2(re-test)		1.000	0.526	0.472
M3(re-test)			1.000	0.481
M4(re-test)				1.000

## Supplementary Materials

The following are available online at https://www.mdpi.com/2227-9032/8/3/318/s1, SCALA MARX (ARS), MARX SCALE (ENGLISH VERSION).
