# Supplementary material for: Cross-Cultural Adaptation and Validation of the Romanian Marx Activity Rating Scale for Anterior Cruciate Ligament Reconstruction
Source: Healthcare (Basel). 2020 Sep 4;8(3):318. doi: 10.3390/healthcare8030318 (PMC7551582; doi:10.3390/healthcare8030318)
Supplement: Supplementary file 1 [file healthcare-08-00318-s001.pdf]

# SCALA MARX (ARS)

**Instrucţiuni:**
*Indicați cât de des ați efectuat fiecare activitate în starea cea mai sănătoasă și cea mai activă, în ultimul an. Bifați spațiul corespunzător după fiecare element.*

| | Mai puțin de o dată pe lună | O dată pe lună | O dată pe săptămână | De 2 sau 3 ori pe săptămână | De 4 sau mai multe ori pe săptămână |
|---|---|---|---|---|---|
| **Alergare:** alergare în timp ce practicați un sport sau alergare recreațională (jogging) | ○ | ○ | ○ | ○ | ○ |
| **Alergare cu schimbare direcției de mișcare:** schimbarea bruscă a direcției în timp ce alergați | ○ | ○ | ○ | ○ | ○ |
| **Decelerare(oprire):** opriți brusc în timp ce alergați | ○ | ○ | ○ | ○ | ○ |
| **Pivotare:** răsuciți corpul cu piciorul fixat în timpul practicării sportului; De exemplu: schi, patinaj, lovire, aruncare, lovirea unei mingi (golf, tenis, squash) etc. | ○ | ○ | ○ | ○ | ○ |

**MARX SCALE (ENGLISH VERSION)** (Marx et al[6,9])

---

Please indicate how often you performed each activity in your healthiest and most active state, in the past year. Kindly put a mark on the appropriate space after each item.

---

| | Less than one time in a month | One time in a month | One time in a week | 2 or 3 times in a week | 4 or more times in a week |
|---|---|---|---|---|---|
| **Running:** running while playing a sport or jogging | ○ | ○ | ○ | ○ | ○ |
| **Cutting:** changing directions while running | ○ | ○ | ○ | ○ | ○ |
| **Deceleration:** coming to a quick stop while running | ○ | ○ | ○ | ○ | ○ |
| **Pivoting:** turning your body with your foot planted while playing sport; For example: skiing, skating, kicking, throwing, hitting a ball (golf, tennis, squash), etc. | ○ | ○ | ○ | ○ | ○ |
